# Concentrated Preterm Formula as a Liquid Human Milk Fortifier at Initiation Stage in Extremely Low Birth Weight Preterm Infants: Short Term and 2-year Follow-up Outcomes

**DOI:** 10.3390/nu12082229

**Published:** 2020-07-26

**Authors:** Yung-Chieh Lin, Yen-Ju Chen, Chao-Ching Huang, Chi-Chang Shieh

**Affiliations:** 1 Institute of Clinical Medicine, College of Medicine, National Cheng-Kung University, Tainan 70403, Taiwan; drapple@mail.ncku.edu.tw; 2 Department of Pediatrics, National Cheng Kung University Hospital, College of Medicine, National Cheng-Kung University, Tainan 70403, Taiwan; yensweet@gmail.com (Y.-J.C.); huangped@mail.ncku.edu.tw (C.-C.H.)

**Keywords:** extremely low birth weight preterm infant, growth retardation, concentrated formula, liquid fortification, human milk, outcome, follow-up

## Abstract

Human milk (HM) must be accurately fortified for extremely low birth weight (ELBW) preterm infants with human milk fortifiers (HMFs). Powdered HMF has some limitations in terms of sterilization and accuracy. A concentrated preterm formula (CPF) may serve as a safe liquid HMF to facilitate growth. Hence, we launched a quality improvement project for fortification accuracy of minute volume HM. A CPF, Similac Special Care 30 (SSC30), was newly introduced as an HMF when daily feeding reached 100 cm^3^/kg. CPF + HM (1:2 volume ratio), CPF + HM (1:1 volume ratio), and powdered HMF + HM (1 packet in 25 cm^3^) represented three fortification stages. Fortification shifted to powdered HMF while tolerable feeding reached 25 cm^3^/meal. The outcome was compared before (Period-I, January 2015 to June 2016, *n* = 37) and after the new implement (Period-II, July 2016 to December 2017, *n* = 36). Compared with the Period-I group, the Period-II group had significantly higher daily enteral milk intake in the first 4 weeks of life, and higher percentages of fortification in the HM-fed infants in the first 8 weeks after birth. The Period-II group also significantly increased in body weight growth in terms of z-score at term equivalent age (*p* = 0.04) and had better language and motor performance at 24 months old (*p* = 0.048 and *p* = 0.032, respectively). Using the liquid CPF as a strategical alternative fortification of HM might be beneficial for extremely preterm infants in terms of growth as well as neurodevelopment.

## 1. Introduction

Human milk (HM) is the first choice of feeding for preterm infants [1,2,3]. HM has numerous benefits for preterm infants to reduce the risks of diseases, such as necrotizing enterocolitis (NEC) [4,5,6], nosocomial infection, sepsis [7], and bronchopulmonary dysplasia [8]. The consumption of HM is associated with the improvements in neurodevelopmental outcomes in preterm infants [7,9,10].

The nutritional content of unfortified HM, however, can not completely support the growth of preterm infants [11,12]. The literature recommends fortified human milk in infants born before 32 weeks gestation and for certain infants born at 32–36 weeks of gestation [13]. Human milk fortifier (HMF) (24 kcal/oz) is indicated for all breast milk-fed infants weighing less than 2000 g and should be initiated when the infant is tolerating breast milk feeds of 25 cm^3^/day. Infants receiving 25 cm^3^ of breast milk on the first day of feeds should wait until day 3 or 4 of life before starting human milk fortifier [13].

Fortification accuracy is imperative to prevent feeding intolerance and promote optimal health and growth [14] when feeding extremely preterm infants was preferred to be more aggressive and earlier [15]. Methods for accuracy fortification are ever proposed, including fortifying in a pooled donor milk [16] or using liquid HMF [17]. The uncertain osmolarity issue of powdered HMF in pooled human milk remains controversial. Sterile liquid human milk-based fortifier makes fortification possible at the minute volume of 40 cm^3^/kg/day [17].

The expensive concentrated liquid HMF, however, is not available worldwide, and thus liquid concentrated preterm formula (CPF) could be an alternative, one that is available in many countries. Liquid concentrated preterm formula (CPF), as reported, is an alternative to liquid HMF [18,19]. Similac Special Care 30 (SSC30), a CPF, is suitable for use as a human milk fortifier because it increases the nutrient content of human milk without increasing the osmolality. Under specific ratios, the mixture nutrients of SSC30 with HM are close to the powder HMF-fortified HM. Using sterile syringes to take SSC30 also leads to the accuracy of targeting caloric density in extremely preterm infants with small feeding volume.

Hence, our team considered the fact that SSC30 could function as HMF for early fortification and as a bridge before full fortification fortified by powder HMF. In July 2016, our nutritional team launched a quality improvement (QI) project to use SSC30 as the first HMF in tiny HM-fed preterm infants. We found that the use of SSC30 as early fortification may enhance the short-term postnatal growth trajectory and may improve neurodevelopmental outcomes at follow-up in extremely low birth weight (ELBW) preterm infants.

To date, few studies have investigated the follow-up data after the use of SSC30 as an initial fortifier. This study aimed to review the short-term morbidities, postnatal growth, and the long-term neurodevelopment outcome of the ELBW preterm infants after our quality improvement project.

## 2. Materials and Methods

This study utilized a comparative effectiveness research design to identify the difference in short- and long-term growth and neurodevelopmental outcome between preterm infants on human milk fortified by powder to those fortified by the formula at initial fortification stage.

### 2.1. Study Population, Setting, and Nutrition Policy

This study was conducted at a 20-bed tertiary neonatal intensive care unit (NICU) at National Cheng Kung University Hospital in Tainan, Taiwan. Approximately 350 neonates are treated at the unit yearly, including approximately 80 infants with very low birth weight (VLBW). Two neonatologists, two residents, and one nurse practitioner were regularly in charge of the patients. A pediatric nutritionist visited the ward twice weekly. The unit was equipped with a milk preparation room and a donor HM bank supported by Taipei City Human Milk Bank. Almost all ELBW infants were fed with their mothers’ milk or donor milk in our units.

#### 2.1.1. Nutrition Policy in Hospitalization

**Figure 1:**
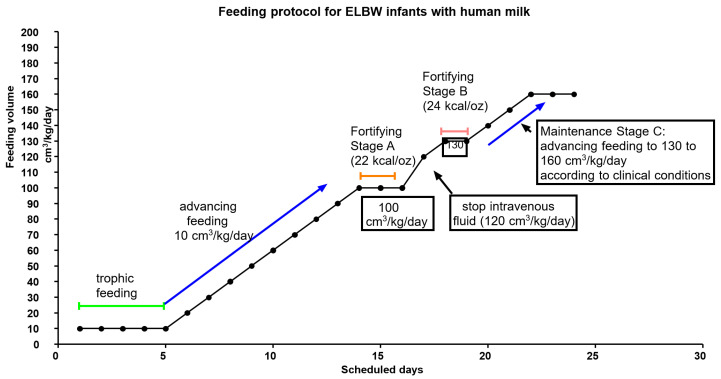
The scheduled feeding protocol for the extremely low birth weight infants.

An overview of the feeding policy since 2015 is displayed in Figure 1. Feeding was recommended on the first day of life, depending on the clinical condition. For ELBW infants, trophic feeding was maintained for 5 days. Trophic feeding was discontinued under critical conditions. The infants were administrated 3.5 g/kg/day of amino acid. Lipid administration was also initiated at 1 g/kg/day once the central line route was established, and this was gradually increased to 3 g/kg/day. After trophic feeding, the ELBW infants received advanced feeding of 10–20 cm^3^/kg/day depending on feeding tolerance.

The goals of each nutrient were set and calculated by the team. The parenteral nutrition weaned when the enteral nutrition advanced. The daily protein intake was controlled at 3.5 g/kg/day, and the lipid intakes were controlled at 3.5 g/kg/day. The daily fluid intake was maintained at 130 to 150 cm^3^/kg/day. The daily calorie was set at 110–120 kcal/kg/day [20,21].

Gastric residuals, bowel conditions, and stool passages were monitored. The feedings were interrupted or workups were implemented if bilious or bloody residuals or massive gastric residuals were observed. Fortification with fortified HM was initiated the day after the feeding amount reached 100 cm^3^/kg/day (Stage A in Figure 1), and intravenous lipid administration was stopped. During fortification, feeding was maintained at 100 cm^3^/kg/day and the condition of the abdomen was monitored. After 2 days of observation, advanced feeding was restarted with fortified milk. Intravenous fluid administration was canceled when the daily fluid intake reached 120 cm^3^/kg/day. The second stage of fortification was initiated the day after feeding reached 130 cm^3^/kg/day. The abdomen condition was monitored closely for 2 days after the second stage fortification started (Stage B in Figure 1). The target of daily fluid intake after 130 cm^3^/kg/day was established by the clinician after evaluation (Stage C in Figure 1). Detail intake and output were usually recorded as ward routines and the growth was monitored continuously. Tailored interventions were necessary. The interventions were discussed by the team during the nutritional run.

Blood tests for hematocrit, ionized calcium, alkaline phosphatase, phosphorus, albumin, blood urea nitrogen, total bilirubin, direct bilirubin, alanine aminotransferase, and aspartate aminotransferase were performed. The anthropometry of stable infants was assessed, including daily body weight, weekly body length, and weekly head circumference. Unstable infant measurements were obtained twice weekly.

#### 2.1.2. Nutrition Policy in the Pre-Discharge Period until 24 months Old 

The infants were discharged when they had a stable body temperature in ambient room temperature, adequate growth velocity under oral feeding, stable oxygen peripheral saturation in room air or low flow nasal cannula, and no apneic episode for 3 days, in addition to having well-prepared parents.

Although the fortification material at the initiation in two periods was different, the fortifications were both with a powder human milk fortifier. The fortification was continued at graduate care unit bedsides, as well as at home after discharge. The cessation of fortification was notified to the family when the bodyweight reached 3.6 kg, or over 25th percentile for corrected age after term equivalent age by clinicians’ evaluation. The post-discharge fortification protocol was recommended by the guideline of the Taiwan Society of Neonatology [20].

After discharge, all infants received fortification of their mothers’ own milk and received monthly follow-ups with palivizumab injection for preventing respiratory syncytial virus infection. The fortification of milk was continued until the cessation criteria. After cessation of fortification, the infants continued to receive their mothers’ milk with iron supplements. If infants lacked mothers’ own milk, commercial post-discharge formula (PDF) was used. The duration of PDF could be used until 9 months after discharge or the bodyweight percentile located with 10–50 percentile for corrected age. Semi-solid supplementary food was introduced when infants reached the corrected age of 4–6 months. Solid food was dominant foods after the corrected age of 1 year old. Commercial formula or cow’s milk was encouraged as a supplemental source of protein, calcium, and vitamins A and D [22,23].

### 2.2. QI Project

The QI project was launched universally after team discussion in July 2016 to solve the fortification problem and mitigate extra-uterine growth retardation (EUGR) in infants with ELBW in our units [24]. Our team reviewed the problems of separating the package of HMF into an extremely small packet of powder, the growth of the infants, and the possible contaminated process. Experiences were shared, and references were reviewed. The team decided to centralize the fortification with liquid SSC30. A new fortification strategy was established with SSC30 as the initial fortifier at Stage A.

Details of the fortification protocol and the comparison of selected nutrients are listed in Table 1.

Previously, Stage A fortification used the F1 protocol (22 kcal/oz), and Stage B fortification used the F2 protocol (24 kcal/oz). The fortification was conducted at patients’ bedsides.

In the new project, Stage A fortification used the F3 protocol (23 kcal/oz), and Stage B fortification used the F4 protocol (25 Kcal/oz). The preparation of milk was centralized in a cleanroom. When the infants could tolerate 25 cm^3^ per meal, the fortification was shifted to F2 at patients’ bedsides. For the QI project, a user-friendly website (http://ped.hosp.ncku.edu.tw/Feeding/index.htm) was designed for resident doctors and nurse practitioners to select the correct order on the basis of bodyweight. The website also directed the time of changing from CPF to powdered HMF.

### 2.3. Follow-Up Program of NICU Graduates

Since 1995, infants with ELBW have been followed-up from discharge to the age of 2 years [26,27]. At the follow-up clinic, the team performed health evaluation; physical and neurological examinations; anthropometry; and neurodevelopment assessment by the Bayley Scales of Infant Development, third edition (BSID-III). BSID-III evaluated the three domains of function: motor, language, and cognition of the preterm infants. The abnormal score was defined score of less than 85 [28].

### 2.4. Study Design

This was a retrospective and longitudinal study to review the outcomes of the QI project. This study was approved by the Institutional Review Board of National Cheng Kung University Hospital (B-ER-107-216) and (ER-98-135).

All ELBW preterm infants born between January 2015 and December 2017 were enrolled and separated into two groups: Period-I group, and Period-II group, on the basis of the fortification policy before and after the QI project implementation. Infants with congenital disease, surgical abdomen diseases, and severe intraventricular hemorrhage, which affected the growth and neurological outcome, were excluded. Infants who were simultaneously exposed to the two fortification protocols were excluded from this study. Mortality and infants transferred to other hospitals were also excluded.

The variables collected perinatal history, the day of nutrition intervention after admission, length of hospital stay, anthropometrical measurement (body weight, body length, and head circumference), medication history, and neonatal morbidities during hospitalization such as surfactant-treated respiratory distress syndrome, patent ductus arteriosus, and metabolic bone disease. The gestational age of a fetus was calculated from the established date of delivery according to the last menstrual period. If the biometric ultrasound measurements varied from the menstrual dates by more than 5 to 7 days in the first trimester, we used ultrasound to establish the date of delivery was used.

The primary outcome data were collected at term equivalent age (anthropometry) and at the corrected age of 24 months (anthropometry and neurodevelopment). We calculated the z-scores and percentiles on the basis of reference data [29] on the website (https://apps.cpeg-gcep.net/premZ_cpeg/).

The secondary outcome was the major neonatal morbidity during hospitalization [30], including treated retinopathy of prematurity (ROP), necrotizing enterocolitis (NEC) stage 2 or 3 [31], chronic lung disease defined by the criteria of National Institute of Health [32], culture-proven sepsis, and periventricular leukomalacia.

### 2.5. The Statistics and Analysis

This study was observational, and therefore the study population was not recruited on the basis of the statistical power calculation. All analyses were performed using SPSS (Version 26, IBM, Armonk, NY, USA). A *p*-value of 0.05 was considered significant. Continuous variables were compared using Student’s *t*-test or Mann–Whitney’s *U* test, whereas categorical data were compared using the chi-square test or Fisher’s exact test, where applicable. The outcome of infants was compared between the study periods utilizing the analysis of covariance, with a priori covariates.

## 3. Results

**Figure 2:**
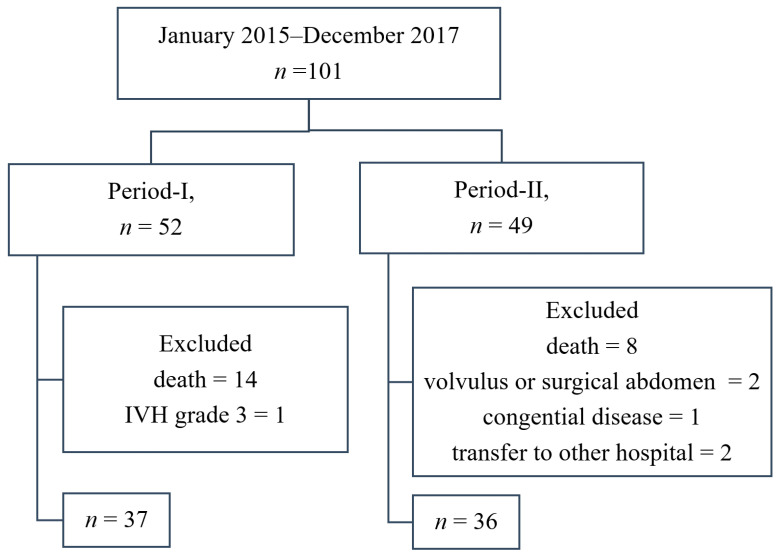
Flowchart of the study periods and participants. IVH: intraventricular hemorrhages.

From January 2015 to December 2017, 103 extremely preterm infants were admitted to our NICU. After excluding two patients whose hospitalization overlapped the two periods, we enrolled 101 patients in total. The enrollment flowchart is displayed shown in Figure 2. These 101 infants were separated into two groups: before (Period-I) and after (Period-II) the project implementation. After exclusion, the Period-I group comprised 37 infants, and the Period-II group comprised 36 infants. The overall survival rate was 73% in Period-I and 83% in Period-II. The survival rates did not differ significantly.

The demographics, perinatal complications, and neonatal complications of the infants in Period-I and Period-II groups are listed in Table 2. Infants in both groups were comparable in gestational age, gender, multiple pregnancies, small for the gestational age, body weight, body length, and head circumference at birth, and the rates of neonatal morbidities such as severity of respiratory distress syndrome, hemodynamically significant patent ductus arteriosus, and sepsis. Infants in Period II had relatively lower gestational ages, which were reflected by the significantly lower Apgar’s scores at both 1 min and 5 min.

Table 3 summarizes the nutritional management of the two groups, including the initial management, the initial fortification, and the summarized data before the postmenstrual age (PMA) of 36 weeks. The two groups were similar in the age at initial nutrition management, namely, the time to start the first feeding; the age at the use of parenteral nutrition or lipid infusion; and the total days under parenteral nutrition, lipid infusion, and enteral feeding before the postmenstrual age of 36 weeks. The Period-II group was significantly smaller at the postmenstrual age of having the first fortification compared to the Period-I group. Lipid infusion was marginally significantly earlier in Period-II (*p* = 0.06). However, the different durations and effects attributed to overall nutrition were considered minimal.

**Figure 3:**
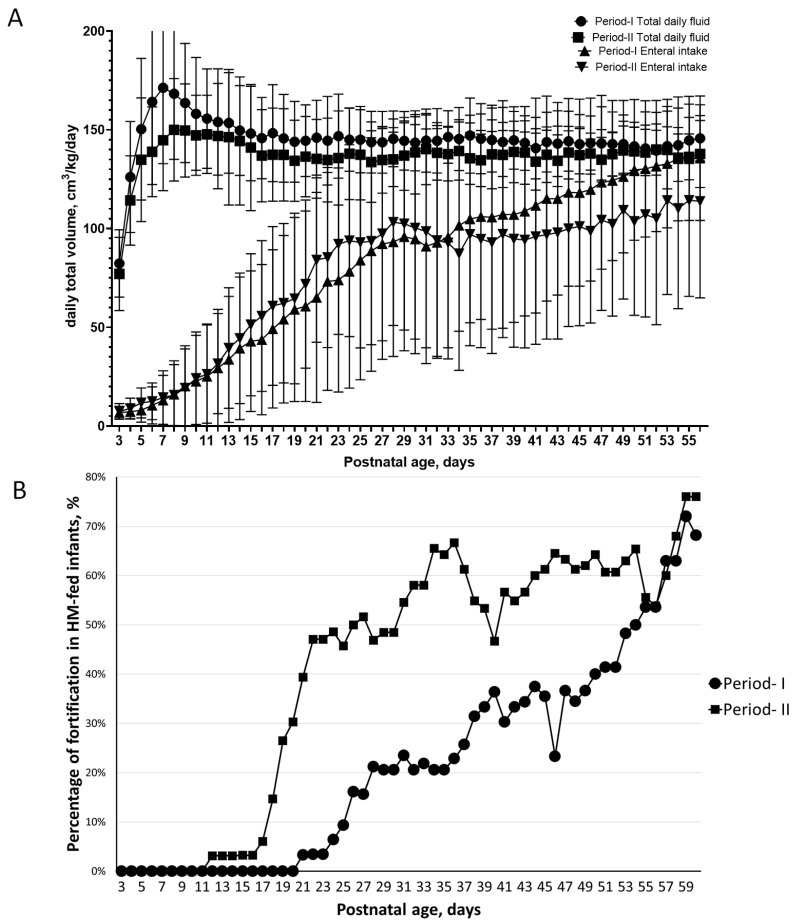
The dynamics of daily intakes and enteral intake, and the fortification percentage in the first two months of life between the extremely low birth weight (ELBW) preterm infants in Period-I and Period-II. (**A**) The daily fluids intake and daily milk in the two study groups. (**B**) Daily percentage of infants receiving fortified human milk (HM) among infants receiving HM in the two periods.

The daily fluid intakes, enteral intakes, and percentages of fortification in HM-fed infants in the first two months of life between the two periods groups are illustrated in Figure 3. The first two months are illustrated because preterm infants were then moved from the NICU to graduate care units. The recordings in the NICU from the electronic system are reliable.

Figure 3A illustrates the daily fluid intake and daily milk intake in the first 2 months of life. In both periods, infants started to receive fortified-HM at around the 30th day of postnatal age, a 2-week delay as the scheduled protocol. In the first 2 months after birth, the average daily fluid intake (mean ± SD) was significantly higher in the Period-I group (143.5 ± 25 cm^3^) than that in the Period-II group (136 ± 23 cm^3^) (*p* 0.001). In the first month of life, the average daily enteral milk intake (mean ± SD) was significantly higher in the Period-II group (56.1 ± 50.1 cm^3^) than that in the Period-I group (49.0 ± 51.0 cm^3^) (*p* = 0.002). In the second month of life, however, the average daily enteral milk intake (mean ± SD) was significantly higher in the Period-I group (116.8 ± 48 cm^3^) than that of the Period-II group (101 ± 52 cm^3^) (*p* 0.001). These findings indicate that infants in Period-II had earlier feeding but lower daily fluid intake.

The percentage of infants receiving fortified HM among infants receiving HM in Period-II clearly increased after the 19th day of life in the Period-II group compared to that in the Period-I group (*p* = 0.002; Figure 3B). On the 27th day of life, more than 50% of HM-fed infants in the Period-II group were receiving fortification compared to that of 20% in the Period-I group. The percentage remained significantly higher in infants in Period-I than in infants in Period-II by the 48th day of life (*p* = 0.043). Infants in Period-II were fed faster and received more fortified HM than those in Period-I.

**Figure 4:**
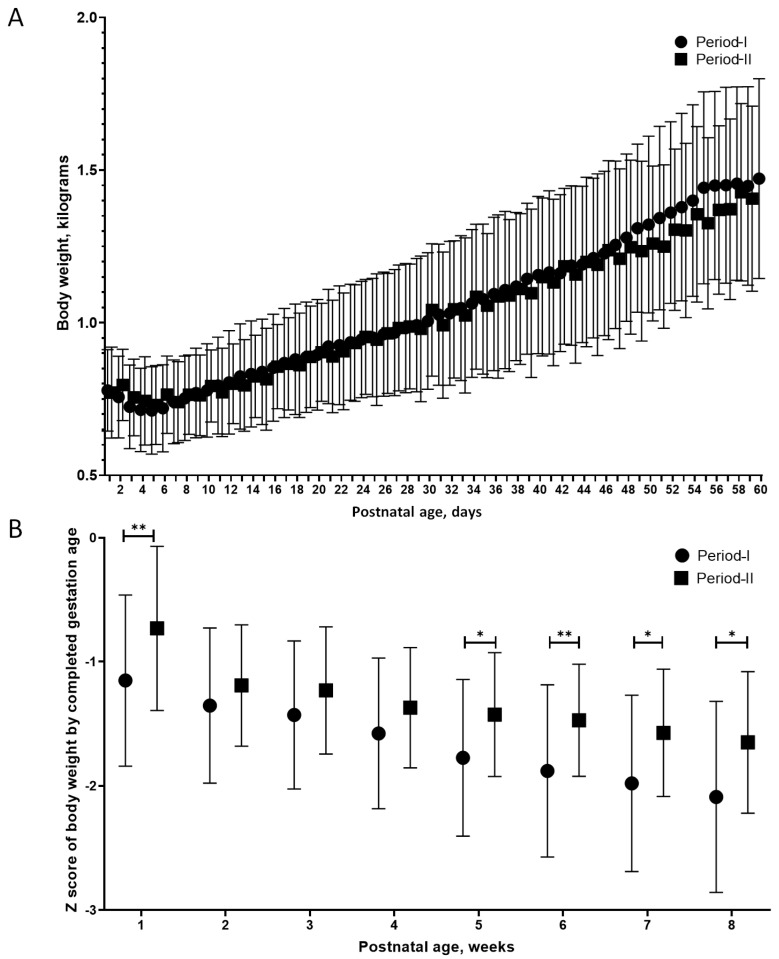
The differences in body weight growth dynamics in the first 2 months of life between the ELBW preterm infants in Period-I and Period-II. (**A**) The daily growth trajectory of infants in the two groups. (**B**) Weekly body weight z-scores by completed gestational age in the two groups. Data interval expressed as mean ± standard deviation. * represented as *p* 0.05; ** represented as *p* 0.01.

Figure 4A illustrates the bodyweight trajectory during the first 2 months of life in two groups. The trajectory curves were very similar. No statistical differences in daily body weight were identified between the two periods. However, infants in Period-II had a relatively younger gestational age (Table 1), and thus we examined the weekly z-score trajectory illustrated in Figure 4B. Comparing the bodyweight z-score of postmenstrual age, we found that infants in the Period-II group had steadily significantly higher z-scores at week 5, week 6, week 7, and week 8 (*p* = 0.01, 0.005, 0.01, and 0.02, respectively) compared to infants in the Period-I group.

Table 4 summarized the clinical course and outcome variables at discharge and the anthropometry at term equivalent age. Infants in Period-II had fewer days of diuretic use, which may be caused by the lower fluid intake compared with the infants in Period-I. The neonatal morbidities were similar between the two groups, and there was no significant difference. The body weight at term equivalent age was significantly higher in infants in Period-II than in infants in Period-I (*p* = 0.04, adjusted for evaluated PMA, gestation age, and sex).

Table 5 summarizes the anthropometry and neurodevelopmental outcomes at the corrected age of 24 months. After adjustment with gestational age at birth and the z-score birth bodyweight, both groups were comparable in terms of body weight, body length, and head circumference at 24 months of age. In contrast, the Period-II group had significantly better performance in the language and motor composite scores by the BSID-III than the Period-I group (*p* = 0.048 and *p* = 0.032, respectively). The percentages of abnormal scores in the three domains were similar in two periods.

## 4. Discussion

In July 2016, we implemented new protocols for fortification and aimed to mitigate postnatal growth retardation in infants with ELBW. In the new protocol, we universally used a CPF as an initial fortifier to facilitate fortification and temporarily resolve the difficulties associated with handling powder HMF. The purpose of this study was to review the outcomes of the implementation of new protocols for fortification.

The outcome of this project was not inferior to the traditional protocol with powder fortification. Furthermore, the fortification strategy in this study displayed certain advantages, including earlier introduction of fortification and higher bodyweight growth at term equivalent age. We also observed a lower fluid load and shorter duration of diuretic administration, better body weight growth by term equivalent age, and improved language and motor composite scores at the age of 24 months. These findings suggest that serving SSC30 as a liquid HMF at the initiation stage of feeding might be beneficial in the postnatal body weight growth trajectory and neurodevelopmental outcome in ELBW preterm infants.

### 4.1. Overall Nutrition Management in Extremely Preterm Infants

In both periods, we started early parenteral nutrition at the first day of life after birth. Our early nutrition policy was compatible with current suggestions [21,33]. Our infants, however, started the lipid infusion later in both periods than recommended. The lipid infusion allowed us to start soon after birth in this study [20]. However, the initiation was delayed when central venous routes were unavailable, or the lipid infusion was incompatible with other infusions in one route, critical conditions, or suspected sepsis.

The feeding initiation time was delayed as the scheduled protocol. The delay might be that the statistic only included the start of tube feeding. Oral colostrum or donor milk swabs [34] were not included. The scheduled protocol was compatible with the publication “Recommendation on Nutritional Care of Taiwan Preterm Infants” issued by the Taiwan Society of Neonatology [20]. However, the difference between the scheduled protocol and the actual start of fortification depended on the clinical condition and the incremental rates. The study subjects were extremely preterm infants, of which 52% of infants of this study were gestational age less than 27 weeks. Feeding progress or milestone were delayed due to clinical morbidity, feeding tolerance, or medications impacting gastrointestinal motility [35,36]. However, recent studies [15,37] have reported early aggressive feeding was safe in ELBW infants. The feeding rate in ELBW infants appeared to be more aggressive and earlier. Hence, our new strategy might be helpful for early aggressive feeding since the feeding volume reaching the fortification may be small.

### 4.2. Feeding and Fortification in Extremely Preterm Infants

Enteral feeding in the clinical care of extremely preterm infants is always challenging for clinicians in the NICU. Under the same feeding protocol, Figure 3A shows that the date of reaching 100 cm^3^/kg/day feeding strategy was very similar in the two periods. Figure 3B, however, shows different fortification patterns between the two periods. Liquid fortification may be easier to commence for clinicians. In Period-I, 70% of HM-fed infants received fortification under 130 cm^3^/kg/day. Our data assumed the clinician chose to advance feeding first and delay the fortification. We were uncertain as to whether the powder HMF had more gastrointestinal intolerance or was difficult to handle. SSC30 was reported as a rescue for infants with intolerance to powder HMF [19]. The osmolarity of SSC30 remains nearly isotonic at 310 mOsm/kg, which was much lower than a pooled fortified donor milk 410.1 ± 27 mOsm/kg at 6 h [16]. Numerous fortification strategies have been proposed [11,21,38]. Traditionally, fortification is initiated when the feeding amount reaches 100 cm^3^/kg/day, and the trend moves towards initiating treatment when daily feedings reach 50–80 cm^3^/kg/day. Hence, the safety of fortified HM in 50-80 cm^3^/kg/day should receive more concern since the intestine of this may be immature.

The accessibility of HM is also important for ELBW infants. In our study, the ELBW infants in both study periods were all exclusively breast milk-fed, with milk provided by a national donor milk distribution site in our hospital [39]. Pasteurized donor milk is transferred to our site every other week, which makes HM easily accessible to the preterm infants in the NICU. In addition, we have a central milk preparation room to share the donor HM and mix with the preterm formula. These factors were fundamental to the success of this study. Our strategy and results provide a practical and operable method to fortify HM during the transition period.

However, the results only demonstrated significant improvements in body weight growth during the transition period and at term equivalent age. The term-equivalent age data for Period-II revealed that the average body weight was 2611 ± 538 g and the z-score was −1.83 ± 1.2 (data not shown). These findings indicate that fortification may remain inadequate and requires multidisciplinary intervention.

### 4.3. The Problem of Powder Form Fortification

Powdered HMFs have been marketed for a long time [40,41]. Powdered additives are easy to use for parents and nursing staff when adding a whole packet to 25 or 50 cm^3^. However, using powdered fortification at the bedside for infants with ELBW has numerous limitations. First, accurately separating packets into 1/8 or 1/4 is difficult. Second, the lack of sterility and the risk of contamination before opening is a concern. Reports have even indicated that powdered additives do not alter the biochemical effect of HM. Furthermore, there are no formal recommendations regarding the timing of adding fortifiers to HM before feeding. The general recommendation is immediately before feeding. Therefore, the mixture should be prepared at the bedside, where cross-contamination could occur. The second problem is the accuracy of fortification. For small volume division, a gram scale should be used in the preparation room, and the mixture would need to be sent to the bedside immediately [25]. However, preparing accurate powder packets could be a heavy workload and carries a risk of contamination.

We believed the difficulties in preparation of small volumes of standard strength fortified in ELBW infants are less reported. We searched the Internet and PubMed. The fortification in the minute amounts of milk is reported in resource-limited settings [42]. We also found a new report [43] in the United Kingdom. This new report developed another method to solve this problem, which was the same as our condition.

We also studied the data in Table 3 and Figure 3. Infants in Period-II did not have significantly longer duration of parenteral nutrition, nor did they have significantly longer feeding duration by PMA at 36 weeks. We suspected that clinicians compensated for feeding infants more unfortified milk, with the volume leading to early cessation of PN in Period-I. This might have contributed to the stunted growth in term equivalent age in Period-I.

### 4.4. The Advangtage and Disadvantage of the Alternatives of Liquid Fortifier

For the safety and feasibility of using CPF to fortify the HM in ELBW infants, our group agreed on the applicability of mixing. We observed that preparing fortification with powder HMF at the bedside was a labor-intensive work for the nurse staff, also having the risk of infection and inaccuracy. Although our study was not the first to use the liquid preterm formula as a HMF [19,44], this quality improvement project was emphasized as the "transitional use" of the liquid preterm formula to overcome the problem of powder HMF. Using CPF as an HMF in the early stage of fortification might accelerate the postnatal growth trajectory.

Using liquid preterm formula as a fortification clinically may be confronted with the problem of decreases in total human milk intakes since the preterm formula is not as concentrated as the commercial liquid HM. Fortifying HM with liquid CPF may decrease the amount of HM intake, but it can increase the total calories and intake protein amounts. A complete replacement of powder HMF with CPF was considered unethical and inappropriate, but it might be acceptable as “transient” replacement therapy.

Using the liquid CPF as an HMF, our study showed that the percentage of fortification in HM-fed ELBW infants reached above 50% at the third postnatal week. The following powder fortification remains important when the preterm infants grow up and tolerate 25 cm^3^ per meal. It is appropriate to shift back full fortification with powder HMF to maximize the human milk consumption when the full feeding volume reached 25 cm^3^ per meal. Our clinical trial of using the three-stage fortification strategy, which was tailored-made for ELBW preterm infants, showed significantly beneficial effects in the postnatal growth at term equivalent age and neurodevelopmental outcome at follow-up when compared with the historical controls.

### 4.5. Additional Benifits of Maintaining HM Use in Preterm Infants

HM is unique for human infants. In addition to the immunologic protecting factors provided by maternal secretor immunoglobulin, HM provides special HM oligosaccharides (HMOs) and numerous crucial microbiota. HMOs, which are a prebiotic, and microbiota protect infants with ELBW from NEC. Increasing evidence has confirmed the theory of the gut–brain axis, in which intestine health may affect the developing brain [45]. The intestinal microbiome differs between infants exclusively receiving formula and receiving HM, which affects human health [46,47,48]. Our strategic fortification provides a balance between higher nutrients from formula and reserving the benefit of HM under the shortage of commercial liquid HMF. However, the effects of our protocol on the microbiota should be investigated.

### 4.6. Limitations and Strengths of This Study

This study had several limitations, including the small sample size and the lack of data regarding the exact daily caloric intake. The small sample size of ELBW preterm infants enrolled in the trial, as well as the use of historical controls, are of concern. The sample size was not computed. Nonetheless, there was a trend that the revised regimen was non-inferior to the conventional approach. The exact caloric intake might be less affected because every preterm infant’s nutrition was tailor-made and prescribed through detailed calculation daily by the physicians. The high follow-up rate (84.9%) was a strength. A neurodevelopmental follow-up assessment is critical to evaluate the effectiveness of the early-life nutritional intervention in the developing immature brain of ELBW preterm infants.

## 5. Conclusions

Using the concentrated liquid preterm formula as a strategical alternative fortification of HM at the initial stage of fortification is feasible. It might be beneficial for ELBW preterm infants in improving postnatal growth and neurodevelopmental outcome. A further randomized trial with control potential confounding variables should be conducted in future.

## Figures and Tables

**Table 1:** Comparison of the nutrient contents of powdered human milk fortifier (HMF) and concentrated preterm formula (CPF) mixed with human milk under different mixing protocols.

Protocol ID	F1	F2	F3	F4
Type	Powder HMF	Powder HMF	CPF	CPF
Dilution/mixing	2 packets to 100 cm^3^ HM	4 packets to 100 cm^3^ HM	SSC30 with HM, 1:2 volume ratio	SSC30 with HM, 1:1 volume ratio
Per 100 cm^3^ of mixed milk				
Energy, kcal	73.1	79	79	84.3
Protein, g	1.84	2.34	2	2.23
Iron, mg	0.292	0.458	0.7	0.97
Ca, mg	82.2	138.1	7	103.7
P, mg	45.6	77.7	42	57.1
Vitamin D, IU	61	119	52	77
Osmolality, mOsm/kg H_2_O	297	329	302	310
Place of mixture	bedsides	bedsides	central clean room	central clean room

Nutrient values adapted from published literature [19,25,18].

**Table 2:** The demographic, perinatal, and neonatal data of the two study groups.

	Period-I	Period-II	*p*-Value
Numbers of patients	37	36	
GA (mean ± SD), week (range)	27.3 ± 2 (23.5–30.9)	26.4 ± 2.1 (22.8–31.7)	0.08
BBW (mean ± SD), g (range)	783.1 ± 131.2 (465-978)	773.9 ± 143.7 (478-996)	0.78
BHC (mean ± SD), cm (range)	23.5 ± 1.6 (18.5–25)	23.1 ± 1.7 (19.5–25.5)	0.32
BBL (mean ± SD), cm (range)	33.3 ± 2.4 (26–36)	32.7 ± 2.9 (26.8–41)	0.38
Z-score of BBW	−0.81 ± 0.83	−0.49 ± 0.75	0.09
Z-score of BHC	−0.77 ± 0.8	−0.81 ± 0.71	0.85
Z-score of BBL	−0.82 ± 1.16	−0.96 ± 2.64	0.77
Male, *n* (% of total)	20 (54)	17 (47)	0.20
CS, *n* (% of total)	10 (27)	11 (35.6)	0.80
SGA, *n* (% of total)	10 (27)	5 (13.8)	0.17
Multiple pregnancy *n* (% of total)	1 (2.7)	2 (5.6)	0.54
Antenatal steroid, *n* (% of total)	36 (97.3)	36 (100)	0.32
APGAR scores			
1-min	5.3 ± 1.7	4.3 ± 2.1	0.02
5-min	7.5 ± 1.4	6.5 ± 2.2	0.02
pH of 1st blood gas	7.27 ± 0.07	7.26 ± 0.16	0.55
Surfactant therapy, *n* (% of total)	17 (45.9)	17 (47.2)	0.53
HsPDA, *n* (% of total)	13 (35.1)	12 (33.3)	0.57
Early sepsis, *n* (% of total)	4 (10.8)	2 (5.5)	0.67

*n*: number, GA: gestational age, BBW: birth bodyweight, BHC: birth head circumference, CS: cesarean section, HsPDA: hemodynamically significant patent ductus arteriosus, SGA: small for gestation age, SD: standard deviation; BBL: birth body length.

**Table 3:** Parenteral and enteral nutritional intervention parameters during hospitalization.

	Period-I *n* = 37	Period-II *n* = 36	*p*-Value
Postnatal age when nutrition management was commenced (mean ± SD), day			
Central catheter insertions	1.7 ± 1	1.7 ± 1.5	0.98
Parenteral nutrition	1 ± 0.1	1.1 ± 0.2	0.55
Lipid infusion	5.3 ± 4.1	3.9 ± 2	0.06
First enteral feeding	4 ± 1.6	4 ± 1.8	0.78
Diet type before fortification	Exclusive BM or DBM	Exclusive BM or DBM	
When 1st fortification was commenced			
Body weight (mean ± SD), g	1051 ± 200	981 ± 232	0.18
Postnatal age (mean ± SD), days	34.2 ± 14.1	30.5 ± 16.5	0.3
Postmenstrual age (mean ± SD), weeks	32 ± 1.8	30.6 ± 2	0.002
Days of the nutritional supplement before PMA 36 weeks (mean ± SD), day			
NPO	7.8 ± 7.6	7.8 ± 7.2	0.99
Lipid infusion	21 ± 10	25.8 ± 15.4	0.11
Parenteral nutrition	25.8 ± 12.2	30 ± 15.3	0.19
Enteral feeding	56.5 ± 13	61.9 ± 14.1	0.1

PMA: postmenstrual age; NPO: nil per os; BM: breast milk; DBM Donor breast milk.

**Table 4:** Neonatal morbidity, outcome, and anthropometry at discharge and at term equivalent age.

	Period-I	Period-II		
			*p*-value	
*N*	37	36	unadjusted	* adjusted
PMA at discharge (mean ± SD), weeks	38.9 ± 2.6	39.17 ± 2.4	0.709	
Length of hospital stay (mean ± SD), days	82.4 ± 27.2	90 ± 28.5	0.247	
Days on diuretics (mean ± SD), days	23.3 ± 32.4	15.7 ± 22.8	0.035	
Duration of mechanical ventilation (mean ± SD), days	9 ± 15.4	8.3 ± 11.4	0.834	
Postnatal steroid treatment, *N* (%)	5 (13.5)	10 (27.8)	0.156	
Neonatal morbidities during hospitalization				
CLD, *N* (%)	11 (29.7)	18 (50)	0.097	
LOS, *N* (%)	9 (24.3)	4 (11.1)	0.221	
Treated ROP, *N* (%)	3 (8.1)	4 (11.1)	0.711	
NEC ≥ stage 2, *N* (%)	6 (16.2)	4 (11.1)	0.736	
Metabolic bone disease, *N* (%)	10 (27)	8 (22.2)	0.787	
Anthropometry at term equivalent age (mean ± SD)				
PMA at evaluation, weeks	38.5 ± 1.1	39.3 ± 1.5	0.003	
Body weight, g	2302 ± 491	2611 ± 538	0.008	0.04
Body length, cm	44.4 ± 2.3	45.8 ± 3.1	0.048	0.36
Head circumference, cm	31.5 ± 1.8	31.9 ± 1.6	0.098	0.30

N: number, PMA: postmenstrual age, CLD: chronic lung disease, LOS: late onset of sepsis, ROP: retinopathy of prematurity requiring intervention, NEC: necrotizing enterocolitis; * adjusted for the z-score of birth bodyweight, gestational age, sex, and PMA.

**Table 5:** Anthropometry and neurodevelopment outcomes at the corrected age of 24 months.

			*p*-Value	
24 Months Old	Period-I	Period-II	Unadjusted	Adjusted
Numbers	30	32		
Anthropometry				
Body weight, kg	10.8 ± 1.3	11.2 ± 1.9	0.318	0.374
Body length, cm	84.7 ± 3	84.5 ± 4	0.688	0.923
Head circumference, cm	46.5 ± 1.5	46.1 ± 1.6	0.375	0.515
BSID-III				
Cognitive scores	85.5 ± 11.8	88.0 ± 10.6	0.394	0.150
Language scores	83.3 ± 12.3	87.3 ± 10.5	0.178	0.048
Motor scores	81.7 ± 14.1	87.5 ± 10.3	0.070	0.032
Cognitive scores 85, *N* (%)	8	9	1.000	
Language scores 85, *N* (%)	14	11	0.438	
Motor scores 85, *N* (%)	11	9	0.589	

BSID Bayley Scales of Infant Development, 3rd edition; adjusted for gestational age and z-score of birth bodyweight.

## References

[1] Agostoni C., Buonocore G., Carnielli V.P., De Curtis M., Darmaun D., Decsi T., Domellof M., Embleton N.D., Fusch C., Genzel-Boroviczeny O. (2010). Enteral nutrient supply for preterm infants: Commentary from the European Society of Paediatric Gastroenterology, Hepatology and Nutrition Committee on Nutrition. J. Pediatr. Gastroenterol. Nutr..

[2] de Halleux V., Pieltain C., Senterre T., Rigo J. (2017). Use of donor milk in the neonatal intensive care unit. Semin. Fetal Neonatal. Med..

[3] Tudehope D.I. (2013). Human milk and the nutritional needs of preterm infants. J. Pediatr..

[4] Pammi M., Suresh G. (2017). Enteral lactoferrin supplementation for prevention of sepsis and necrotizing enterocolitis in preterm infants. Cochrane Database Syst. Rev..

[5] Zhou P., Li Y., Ma L.Y., Lin H.C. (2015). The role of immunonutrients in the prevention of necrotizing enterocolitis in preterm very low birth weight infants. Nutrients.

[6] Buckle A., Taylor C. (2017). Cost and cost-effectiveness of donor human milk to prevent necrotizing enterocolitis: Systematic review. Breastfeed. Med..

[7] Cortez J., Makker K., Kraemer D.F., Neu J., Sharma R., Hudak M.L. (2018). Maternal milk feedings reduce sepsis, necrotizing enterocolitis and improve outcomes of premature infants. J. Perinatol..

[8] Villamor-Martinez E., Pierro M., Cavallaro G., Mosca F., Kramer B.W., Villamor E. (2018). Donor human milk protects against bronchopulmonary dysplasia: A systematic review and meta-analysis. Nutrients.

[9] Stefanescu B.M., Gillam-Krakauer M., Stefanescu A.R., Markham M., Kosinski J.L. (2016). Very low birth weight infant care: Adherence to a new nutrition protocol improves growth outcomes and reduces infectious risk. Early Hum. Dev..

[10] Lucas A., Morley R., Cole T.J., Lister G., Leeson-Payne C. (1992). Breast milk and subsequent intelligence quotient in children born preterm. Lancet.

[11] Brune K.D., Donn S.M. (2018). Enteral feeding of the preterm infant. NeoReviews.

[12] Hay W.W., Ziegler E.E. (2016). Growth failure among preterm infants due to insufficient protein is not innocuous and must be prevented. J. Perinatol..

[13] Arslanoglu S., Boquien C.-Y., King C., Lamireau D., Tonetto P., Barnett D., Bertino E., Gaya A., Gebauer C., Grovslien A. (2019). Fortification of human milk for preterm infants: Update and recommendations of the European Milk Bank Association (EMBA) Working Group on human milk fortification. Front. Pediatrics.

[14] Steele C. (2018). Best practices for handling and administration of expressed human milk and donor human milk for hospitalized preterm infants. Front. Nutr..

[15] Dorling J., Abbott J., Berrington J., Bosiak B., Bowler U., Boyle E., Embleton N., Hewer O., Johnson S., Juszczak E. (2019). Controlled trial of two incremental milk-feeding rates in preterm infants. N. Engl. J. Med..

[16] Piemontese P., Liotto N., Menis C., Mallardi D., Tabasso C., Perrone M., Bezze E., Plevani L., Roggero P., Mosca F. (2020). Effect of target fortification on osmolality and microbiological safety of human milk over time. J. Pediatr. Gastroenterol. Nutr..

[17] Sullivan S., Schanler R.J., Kim J.H., Patel A.L., Trawoger R., Kiechl-Kohlendorfer U., Chan G.M., Blanco C.L., Abrams S., Cotten C.M. (2010). An exclusively human milk-based diet is associated with a lower rate of necrotizing enterocolitis than a diet of human milk and bovine milk-based products. J. Pediatr..

[18] (2011). Neofax 2011.

[19] Pillai A., Albersheim S., Matheson J., Lalari V., Wei S., Innis S.M., Elango R. (2018). Evaluation of a concentrated preterm formula as a liquid human milk fortifier in preterm babies at increased risk of feed intolerance. Nutrients.

[20] Taiwan Society of Neonatology (2015). Recommendation on Nutritional Care of Taiwan Preterm Infants.

[21] Su B.H. (2014). Optimizing nutrition in preterm infants. Pediatr. Neonatol..

[22] Health Promotion Administration, Ministry of Health and Welfare (2017). Children’s Health Booklet.

[23] American Academy of Pediatrics (2019). Caring for Your Baby and Young Child: Birth to Age 5.

[24] Lin Y.C., Lin Y.J., Lin C.H. (2011). Growth and neurodevelopmental outcomes of extremely low birth weight infants: A single center’s experience. Pediatr. Neonatol..

[25] Abbott Nutrition (2016). Pediatric Nutrition Product Guide 2016.

[26] Wang L.W., Lin Y.C., Tu Y.F., Wang S.T., Huang C.C., Taiwan Premature Infant Developmental Collaborative Study Group (2017). Isolated cystic periventricular leukomalacia differs from cystic periventricular leukomalacia with intraventricular hemorrhage in prevalence, risk factors and outcomes in preterm infants. Neonatology.

[27] Wang L.W., Lin Y.C., Wang S.T., Huang C.C., Taiwan Premature Infant Developmental Collaborative Study Group (2018). Identifying risk factors shared by bronchopulmonary dysplasia, severe retinopathy, and cystic periventricular leukomalacia in very preterm infants for targeted intervention. Neonatology.

[28] Younge N., Goldstein R.F., Bann C.M., Hintz S.R., Patel R.M., Smith P.B., Bell E.F., Rysavy M.A., Duncan A.F., Vohr B.R. (2017). Survival and neurodevelopmental outcomes among periviable infants. N. Engl. J. Med..

[29] Fenton T.R., Kim J.H. (2013). A systematic review and meta-analysis to revise the Fenton growth chart for preterm infants. BMC Pediatr..

[30] Anderson J.G., Baer R.J., Partridge J.C., Kuppermann M., Franck L.S., Rand L., Jelliffe-Pawlowski L.L., Rogers E.E. (2016). Survival and major morbidity of extremely preterm infants: A population-based study. Pediatrics.

[31] Bell M.J., Ternberg J.L., Feigin R.D., Keating J.P., Marshall R., Barton L., Brotherton T. (1978). Neonatal necrotizing enterocolitis. Therapeutic decisions based upon clinical staging. Ann. Surg..

[32] Ehrenkranz R.A., Walsh M.C., Vohr B.R., Jobe A.H., Wright L.L., Fanaroff A.A., Wrage L.A., Poole K., National Institutes of Child Health and Human Development Neonatal Research Network (2005). Validation of the National Institutes of Health consensus definition of bronchopulmonary dysplasia. Pediatrics.

[33] Ziegler E.E. (2011). Meeting the nutritional needs of the low-birth-weight infant. Ann. Nutr. Metab..

[34] Zhang Y., Ji F., Hu X., Cao Y., Latour J.M. (2017). Oropharyngeal colostrum administration in very low birth weight infants: A randomized controlled trial. Pediatr. Crit. Care Med..

[35] Park J., Knafl G., Thoyre S., Brandon D. (2015). Factors associated with feeding progression in extremely preterm infants. Nurs. Res..

[36] Jadcherla S.R., Wang M., Vijayapal A.S., Leuthner S.R. (2010). Impact of prematurity and co-morbidities on feeding milestones in neonates: A retrospective study. J. Perinatol..

[37] Salas A.A., Li P., Parks K., Lal C.V., Martin C.R., Carlo W.A. (2018). Early progressive feeding in extremely preterm infants: A randomized trial. Am. J. Clin. Nutr..

[38] Hsiao C.C., Tsai M.L., Chen C.C., Lin H.C. (2014). Early optimal nutrition improves neurodevelopmental outcomes for very preterm infants. Nutr. Rev..

[39] Chang F.Y., Cheng S.W., Wu T.Z., Fang L.J. (2013). Characteristics of the first human milk bank in Taiwan. Pediatr. Neonatol..

[40] Modanlou H.D., Lim M.O., Hansen J.W., Sickles V. (1986). Growth, biochemical status, and mineral metabolism in very-low-birth-weight infants receiving fortified preterm human milk. J. Pediatr. Gastroenterol. Nutr..

[41] Thompson M., McClead R.E. (1987). Human milk fortifiers. J. Pediatr. Perinat. Nutr..

[42] Kemp J.E., Wenhold F.A.M. (2016). Human milk fortification strategies for improved in-hospital growth of preterm infants. S. Afr. J. Clin. Nutr..

[43] Preparation of Small Volumes (10mL) Standard Strength Fortified M/DEBM. http://swmnodn.org.uk/wp-content/uploads/2020/05/additon-of-HMF-less-than-50ml-EBM-1.pdf.

[44] Willeitner A., Anderson M., Lewis J. (2017). Highly concentrated preterm formula as an alternative to powdered human milk fortifier: A randomized controlled trial. J. Pediatr. Gastroenterol. Nutr..

[45] Carabotti M., Scirocco A., Maselli M.A., Severi C. (2015). The gut-brain axis: Interactions between enteric microbiota, central and enteric nervous systems. Ann. Gastroenterol..

[46] Mueller E., Blaser M. (2018). Breast milk, formula, the microbiome and overweight. Nat. Rev. Endocrinol..

[47] Guaraldi F., Salvatori G. (2012). Effect of breast and formula feeding on gut microbiota shaping in newborns. Front. Cell. Infect. Microbiol..

[48] Ho N.T., Li F., Lee-Sarwar K.A., Tun H.M., Brown B.P., Pannaraj P.S., Bender J.M., Azad M.B., Thompson A.L., Weiss S.T. (2018). Meta-analysis of effects of exclusive breastfeeding on infant gut microbiota across populations. Nat. Commun..

